# Supplementary material for: Prognostic value of the Residual Cancer Burden index according to breast cancer subtype: Validation on a cohort of BC patients treated by neoadjuvant chemotherapy
Source: PLoS One. 2020 Jun 24;15(6):e0234191. doi: 10.1371/journal.pone.0234191 (PMC7313974; doi:10.1371/journal.pone.0234191)
Supplement: S1 File — (PDF) [file pone.0234191.s001.pdf]

SUPPLEMENTAL MATERIAL AND TABLES

Supplementary Material:

Pathological review:

Residual Cancer Burden

To enable the calculation of RCB score, the following variables were retrieved: diameter of the primary tumor bed in the resection specimen (d_{prim}), the proportion of the primary tumor bed that contains invasive carcinoma (f_{inv}), the number of axillary lymph nodes containing metastatic carcinoma (LN), and the diameter of the largest metastasis in an axillary lymph node (d_{met}). Largest bidimensional measurements of the residual primary tumor bed were recorded from the macroscopic description in the pathology report. If multiple tumors were present, the dimensions of the largest were considered. The proportion of invasive carcinoma (finv) within the cross sectional area of the primary tumor bed was estimated from the overall percent area of carcinoma (%CA) and then corrected for the component of in situ carcinoma (%CIS): f_{inv} (1 – (%CIS/100)) (%CA/100).

RCB was calculated with the formule: $RCB = 1.4(f_{inv}\, d_{prim})^{0.17} + [4\,(1 - 0.75^{LN})\, d_{met}]^{0.17}$

Lymphovascular invasion

Presence or absence of LVI was determined by unstained standard formalin-fxed parafn-embedded examination on surgical specimen. Immunostaining with vascular markers was occasionally performed to rule out invasive carcinoma with shrinkage artifact. LVI data were extracted from pathology records by two independent researchers (TL, ASH), and were dichotomized into a binary variable (Post-NAC LVI: yes/ no). Patients whose tumor reached pCR were considered as having no LVI. Results were crosschecked and a breast expert pathologist (ML) resolved discrepancies.

Tumor infiltrating lymphocytes

In accordance with the recommendations of the international TILs Working Group(Dieci et al., 2018), we checked for presence of a mononuclear cell infiltrate in the stroma on hematoxylin and eosin-stained sections without additional staining, after excluding areas around ductal carcinomas in situ (DCIS), and tumor zones with necrosis and artifacts. Infiltrates were scored on a continuous scale, as the mean percentage of the stromal area occupied by mononuclear cells. After NAC, we assessed TIL levels within the borders of the residual tumor bed, as defined by the RCB index(Symmans et al., 2007b). In cases of pCR, the scar area was measured on macroscopic examination. The scar appeared as a white area in the breast parenchyma corresponding to the tumor bed modified by NAC. It was characterized by the presence of histiocytes, lymphocytes, macrophages, fibrosis and elastosis. The whole fibro-inflammatory scar was evaluated on HE sections (size in mm and stromal TIL level evaluation)

Supplementary tables:

Supplementary Table 1: Association between clinical and pathological pre and post-NAC parameters with relapse-free survival after univariate and multivariate analysis in the luminal population. *p* represents the p-value for the wald test , and p* represents the individual p-value versus reference class. Abbreviations: pCR=pathological complete response; BMI=body mass index ; NST= no special type ; ER=oestrogen receptor; PR=progesterone receptor; RCB=residual cancer burden ; NAC=neoadjuvant chemotherapy; AC=anthracyclines ; LVI=lymphovascular invasion, TILs=tumor infiltrating lymphocytes

Supplementary Table 2: Association between clinical and pathological pre and post-NAC parameters with relapse-free survival (TNBC, univariate and multivariate analysis), *p* represents the p-value for the wald test , and p* represents the individual p-value versus reference class. Abbreviations: pCR=pathological complete response; BMI=body mass index ; NST= no special type ; ER=oestrogen receptor; PR=progesterone receptor; NAC=neoadjuvant chemotherapy; AC=anthracyclines; TILs=tumor infiltrating lymphocytes; RCB=residual cancer burden; LVI=lymphovascular invasion

Supplementary Table 3: Association between clinical and pathological pre and post-NAC parameters with relapse-free survival (HER2-positive population, univariate and multivariate analysis), *p* represents the p-value for the wald test , and p* represents the individual p-value versus reference class. Abbreviations: pCR=pathological complete response; BMI=body mass index ; NST= no special type ; ER=oestrogen receptor; PR=progesterone receptor; NAC=neoadjuvant chemotherapy; AC=anthracyclines; TILs=tumor infiltrating lymphocytes; RCB=residual cancer burden; LVI=lymphovascular invasion

Supplementary Table 4: Association between clinical and pathological pre and post-NAC parameters with overall-free survival (luminal population, univariate and multivariate analysis), *p* represents the p-value for the wald test , and p* represents the individual p-value versus reference class. Abbreviations: pCR=pathological complete response; BMI=body mass index ; NST= no special type ; ER=oestrogen receptor; PR=progesterone receptor; NAC=neoadjuvant chemotherapy; AC=anthracyclines; TILs=tumor infiltrating lymphocytes; RCB=residual cancer burden; LVI=lymphovascular invasion

Supplementary Table 5: Association between clinical and pathological pre and post-NAC parameters with overall-free survival (TNBC population, univariate and multivariate analysis), *p* represents the p-value for the wald test, and p* represents the individual p-value versus reference class. Abbreviations: pCR=pathological complete response; BMI=body mass index ; NST= no special type ; ER=oestrogen receptor; PR=progesterone receptor; NAC=neoadjuvant chemotherapy; AC=anthracyclines; TILs=tumor infiltrating lymphocytes; RCB=residual cancer burden; LVI=lymphovascular invasion

Supplementary Table 6: Association between clinical and pathological pre and post-NAC parameters with overall-free survival (HER2-positive population, univariate and multivariate analysis), *p* represents the p-value for the wald test , and p* represents the individual p-value versus reference class. Abbreviations: pCR=pathological complete response; BMI=body mass index ; NST= no special type ; ER=oestrogen receptor; PR=progesterone receptor; NAC=neoadjuvant chemotherapy; AC=anthracyclines; TILs=tumor infiltrating lymphocytes; RCB=residual cancer burden; LVI=lymphovascular invasion

Supplementary Table 7: Summary of previous studies comparing prognosis according to RCB score after neoadjuvant chemotherapy (NAC). Abreviations: CT stands for clinical trial and RA stands for retrospective analysis. Abbreviations: pCR=pathological complete response; BMI=body mass index ; NST= no special type ; ER=oestrogen receptor; PR=progesterone receptor; NAC=neoadjuvant chemotherapy; AC=anthracyclines; TILs=tumor infiltrating lymphocytes; RCB=residual cancer burden; LVI=lymphovascular invasion

Supplementary Table 1: Association between clinical and pathological pre and post-NAC parameters with relapse-free survival after univariate and multivariate analysis in the luminal population.

p represents the p-value for the wald test , and p* represents the individual p-value versus reference class. Abbreviations: pCR=pathological complete response; BMI=body mass index ; NST= no special type ; ER=oestrogen receptor; PR=progesterone receptor; RCB=residual cancer burden ; NAC=neoadjuvant chemotherapy; AC=anthracyclines ; LVI=lymphovascular invasion, TILs=tumor infiltrating lymphocytes.

Luminal										
				Univariate				Multivariate		
**Variable**	**Class**	**Number**	**Events**	**HR**	**CI**	**p***	***p***	**HR**	**CI**	**p**
Pre-NAC parameters										
**Age (years)**	<45	88	34	1			0.34			
	45-55	82	22	0.68	[0.4 - 1.17]					
	>55	52	19	0.99	[0.56 - 1.74]					
**Menopausal status**	pre	146	43	1			**0.042**			
	post	74	30	1.63	[1.02 - 2.61]					
**BMI**	19≤BMI≤25	121	40	1			0.904			
	<19	18	7	1.12	[0.49 - 2.52]					
	>25	83	28	0.93	[0.57 - 1.51]					
**Tumor size**	T1	10	6	1			0.129			
	T2	160	51	0.42	[0.18 - 0.98]					
	T3	52	18	0.5	[0.2 - 1.26]					
**Clinical nodal status**	N0	79	27	1			0.785			
	N1-N2-N3	142	48	0.94	[0.58 - 1.5]					
**Mitotic Index**	≤22	153	52	1			0.634			
	>22	57	20	1.13	[0.68 - 1.9]					
**Histology**	NST	199	63	1			**0.013**			
	others	23	12	2.2	[1.18 - 4.08]					
**Grade**	I-II	119	40	1			0.779			
	III	97	33	1.07	[0.67 - 1.7]					
**ki67**	<20 %	8	3	1			0.92			
	≥20 %	8	3	0.92	[0.19 - 4.58]					
**PR status**	negative	43	19	1			0.157			
	positive	161	48	0.68	[0.4 - 1.16]					
**TILs**	(continuous)			0,98	[0.96-1.01]		0,193			
Post-NAC parameters										
**Nodal Involvment**	0	78	19	1			**<0.001**	1	**-**	**-**
	1-3	100	30	1.2	[0.68 - 2.14]	0.53		1.17	[0.66 - 2.09]	0.592
	≥4	44	26	2.77	[1.53 - 5.01]	**<0.001**		2.72	[1.49 - 4.94]	**0.001**
**RCB class**	pCR	11	3	1			0.142			
	RCB-I	18	2	0.37	[0.06 - 2.23]	0.277				
	RCB-II	109	33	1.13	[0.35 - 3.69]	0.839				
	RCB-III	84	37	1.58	[0.49 - 5.14]	0.446				
**NAC regimen**	AC	3	2	1			0.647			
	AC-Taxanes	202	68	0.51	[0.13 - 2.1]					
	others	17	5	0.5	[0.1 - 2.57]					
**Post-NAC LVI**	no	130	42	1			0.366			
	yes	67	27	1.25	[0.77 - 2.03]					
**TILs**	(continuous)			0,99	[0.96-1.01]		0,331			

Supplementary Table 2: Association between clinical and pathological pre and post-NAC parameters with relapse-free survival (TNBC, univariate and multivariate analysis).

p represents the p-value for the wald test , and p* represents the individual p-value versus reference class. Abbreviations: pCR=pathological complete response; BMI=body mass index ; NST= no special type ; ER=oestrogen receptor; PR=progesterone receptor; NAC=neoadjuvant chemotherapy; AC=anthracyclines; TILs=tumor infiltrating lymphocytes; RCB=residual cancer burden; LVI=lymphovascular invasion.

TNBC										
				Univariate				Multivariate		
Variable	Class	Number	Events	HR	CI	p*	*p*	HR	CI	p
Pre-NAC parameters										
**Age**	<45	119	41	1			0.732			
	45-55	118	37	0.91	[0.58 - 1.41]					
	>55	82	24	0.82	[0.49 - 1.35]					
**Menopausal status**	pre	191	62	1			0.748			
	post	124	38	0.94	[0.62 - 1.4]					
**BMI**	19≤BMI≤25	176	59	1			0.348			
	<19	16	2	0.35	[0.09 - 1.45]					
	>25	126	41	0.99	[0.67 - 1.48]					
**Tumor size**	T1	27	6	1			0.004	1	-	-
	T2	207	59	1.36	[0.59 - 3.14]	0.477		1,12	[0.47 - 2.65]	0,8
	T3	85	37	2.6	[1.09 - 6.17]	0.03		2,08	[0.86 - 5.02]	0.102*
**Clinical nodal status**	N0	141	42	1			0.293			
	N1-N2-N3	178	60	1.24	[0.83 - 1.84]					
**Mitotic Index**	≤22	124	38	1			0.398			
	>22	177	62	1.19	[0.79 - 1.78]					
**Histology**	NST	291	91	1			0.46			
	others	25	10	1.28	[0.67 - 2.46]					
**Grade**	I-II	40	14	1			0.812			
	III	272	86	0.93	[0.53 - 1.64]					
**Ki67**	<20 %	22	7	1			0.584			
	≥20 %	120	45	1.25	[0.56 - 2.77]					
**NAC regimen**	AC	54	22	1			0.225			
	AC-Taxanes	222	71	0.84	[0.52 - 1.36]					
	Others	43	9	0.51	[0.23 - 1.1]					
**TILs**	(continuous)			0,98	[0.98-0.99]		0,006			
Post-NAC parameters										
**Nodal Involvment**	0	238	52	1			<0.001			
	1-3	49	29	3.72	[2.36 - 5.88]	<0.001				
	≥4	32	21	4.54	[2.73 - 7.56]	<0.001				
**RCB class**	pCR	123	18	1			<0.001	1	-	-
	RCB-I	23	4	1.23	[0.42 - 3.63]	0.711		1,18	[0.4 - 3.53]	0,762
	RCB-II	131	50	3.1	[1.81 - 5.31]	<0.001		2,7	[1.55 - 4.7]	<0.001
	RCB-III	42	30	*9.01*	[4.99 - 16.27]	<0.001		6,77	[3.52 - 13.01]	<0.001
**LVI**	no	232	56	1			<0.001	1	-	-
	yes	55	34	3.5	[2.28 - 5.38]	<0.001		1,57	[1 - 2.47]	0,049
**TILs**	(continuous)			1	[0.99-1.01]		0,98			

* p-value T3 *versus* T2 : 0.003

Supplementary Table 3: Association between clinical and pathological pre and post-NAC parameters with relapse-free survival (HER2-positive population, univariate and multivariate analysis).

p represents the p-value for the wald test , and p* represents the individual p-value versus reference class. Abbreviations: pCR=pathological complete response; BMI=body mass index ; NST= no special type ; ER=oestrogen receptor; PR=progesterone receptor; NAC=neoadjuvant chemotherapy; AC=anthracyclines; TILs=tumor infiltrating lymphocytes; RCB=residual cancer burden; LVI=lymphovascular invasion.

HER2- positive										
				Univariate				Multivariate		
**Variable**	**Class**	**Number**	**Events**	**HR**	**CI**	**p***	***p***	**HR**	**CI**	**p**
Pre-NAC parameters										
**Age (years)**	<45	78	17	1			0.104			
	45-55	54	8	0.63	[0.27 - 1.48]	0.291				
	>55	44	3	0.28	[0.08 - 0.95]	**0.042**				
**Menopausal status**	pre	114	22	1			0.099			
	post	61	6	0.47	[0.19 - 1.15]					
**BMI**	19≤BMI≤25	117	12	1			**0.011**	1	-	-
	<19	7	2	3.16	[0.7 - 14.19]	0.133		6,24	[1.12 - 34.66]	**0,036**
	>25	52	14	3.17	[1.46 - 6.88]	**0.004**		2,88	[1.25 - 6.63]	**0,013**
**Tumor Size**	T1	10	1	1			0.642			
	T2	114	17	1.43	[0.19 - 10.74]					
	T3	52	10	1.97	[0.25 - 15.46]					
**Clinical Nodal Status**	N0	62	9	1			0.695			
	N1-N2-N3	114	19	1.17	[0.53 - 2.59]					
**Mitotic Index**	≤22	112	20	1			0.679			
	>22	59	8	0.84	[0.37 - 1.91]					
**Histology**	NST	170	28	1			0.998			
	other	5	0	0	NA					
**Grade**	I-II	52	16	1			**0.002**	1	-	-
	III	121	12	0.3	[0.14 - 0.63]			0,19	[0.08 - 0.43]	**<0.001**
**ki67**	<20 %	3	0	1			0.999			
	≥20 %	18	3	NA	NA					
**ER status**	negative	77	10	1			0.364			
	positive	99	18	1.43	[0.66 - 3.1]					
**PR status**	negative	111	14	1			0.381			
	positive	60	11	1.42	[0.65 - 3.14]					
**NAC regimen**	AC	4	1	1			0.279			
	AC-Taxanes	152	22	0.64	[0.09 - 4.72]					
	Others	20	5	1.39	[0.16 - 11.96]					
**TILs**	**(continuous)**			0,99	[0.98-1.01]		0,619			
Post-NAC parameters										
**Nodal involvment**	0	129	15	1			**0.04**			
	1-3	39	10	2.16	[0.97 - 4.81]	0.06				
	others	8	3	3.86	[1.11 - 13.4]	**0.033**				
**RCB class**	pCR	68	2	1			**0.002**	1	-	-
	RCB-I	24	1	1.52	[0.14 - 16.81]	0.731		2,72	[0.24 - 31.02]	0,421
	RCB-II	69	19	10.27	[2.39 - 44.11]	**0.002**		7,17	[1.56 - 32.89]	**0,011**
	RCB-III	15	6	16.53	[3.33 - 82.01]	**<0.001**		12,51	[2.36 - 66.27]	**0,003**
**LVI**	no	138	10	1			**<0.001**	1	-	-
	yes	26	14	10.29	[4.55 - 23.27]	**<0.001**		6,24	[2.68 - 14.56]	**<0.001**
**TILs**	(continuous)			1,03	[1.01-1.06]		**0,001**			

Supplementary Table 4: Association between clinical and pathological pre and post-NAC parameters with overall-free survival (luminal population, univariate and multivariate analysis).

p represents the p-value for the wald test , and p* represents the individual p-value versus reference class. Abbreviations: pCR=pathological complete response; BMI=body mass index ; NST= no special type ; ER=oestrogen receptor; PR=progesterone receptor; NAC=neoadjuvant chemotherapy; AC=anthracyclines; TILs=tumor infiltrating lymphocytes; RCB=residual cancer burden; LVI=lymphovascular invasion

Luminal										
				Univariate				Multivariate		
**Variable**	**Class**	**Number**	**Events**	**HR**	**CI**	**p***	***p***	**HR**	**CI**	**p**
Pre-NAC parameters										
**Age (years)**	<45 y.o	88	19	1			0.942			
	45-55 y.o	82	17	1.11	[0.57 - 2.14]					
	>55 y.o	52	10	0.99	[0.46 - 2.13]					
**Menopausal status**	pre	146	24	1			**0.022**	1	-	-
	post	74	20	2.01	[1.1 - 3.64]			2.01	[1.1 - 3.64]	**0.022**
**BMI class**	19≤BMI≤25	121	27	1			0.613			
	<19	18	4	1.11	[0.39 - 3.17]					
	>25	83	15	0.75	[0.4 - 1.4]					
**Tumor size**	T1	10	3	1			0.642			
	T2	160	31	0.63	[0.19 - 2.06]					
	T3	52	12	0.78	[0.22 - 2.8]					
**Clinical nodal status**	N0	79	15	1			0.862			
	N1-N2-N3	142	31	1.06	[0.57 - 1.96]					
**Mitotic Index**	≤22	153	30	1			0.195			
	>22	57	14	1.53	[0.8 - 2.92]					
**Histology**	NST	199	41	1			0.778			
	others	23	5	1.14	[0.45 - 2.9]					
**Grade**	I-II	119	23	1			0.454			
	III	97	22	1.25	[0.7 - 2.25]					
**ki67**	<20	8	1	1			0.361			
	≥20	8	3	2.87	[0.3 - 27.63]					
**PR status**	negative	43	12	1			0.363			
	positive	161	29	0.73	[0.37 - 1.44]					
**NAC regimen**	AC	3	1	1			0.471			
	AC-Taxanes	202	44	1.07	[0.15 - 7.81]					
	Others	17	1	0.31	[0.02 - 4.99]					
**TILs**	(continuous)			0,99	[0.97-1.01]		0,47			
Post-NAC parameters										
RCB	pCR	11	2	1			0.968			
	RCB-I	18	0	0	[0 - Inf]	0.997				
	RCB-II	109	22	1.08	[0.25 - 4.6]	0.918				
	RCB-III	84	22	1.24	[0.29 - 5.32]	0.769				
**Nodal Involvment**	0	78	11	1			**0.048**			
	1-3	100	19	1.23	[0.59 - 2.6]	0.58				
	≥4	44	16	2.42	[1.12 - 5.24]	**0.025**				
**LVI**	no	130	23	1			0.072			
	yes	67	20	1.73	[0.95 - 3.16]	0.072				
**TILs**	(continuous)			0,99	[0.97-1.03]		0,715			

Supplementary Table 5: Association between clinical and pathological pre and post-NAC parameters with overall-free survival (TNBC population, univariate and multivariate analysis).
p represents the p-value for the wald test, and p* represents the individual p-value versus reference class. Abbreviations: pCR=pathological complete response; BMI=body mass index ; NST= no special type ; ER=oestrogen receptor; PR=progesterone receptor; NAC=neoadjuvant chemotherapy; AC=anthracyclines; TILs=tumor infiltrating lymphocytes; RCB=residual cancer burden; LVI=lymphovascular invasion.

TNBC										
				Univariate				Multivariate		
**Variable**	**Class**	**Number**	**Events**	**HR**	**CI**	**p***	***p***	**HR**	**CI**	**p**
Pre-NAC parameters										
**Age (years)**	<45	119	33	1			0.479			
	45-55	118	26	0.79	[0.47 - 1.32]					
	>55	82	17	0.72	[0.4 - 1.3]					
**Menopausal status**	pre	191	48	1			0.596			
	post	124	28	0.88	[0.55 - 1.41]					
**BMI class**	19≤BMI≤25	176	43	1			0.562			
	<19	16	2	0.47	[0.11 - 1.93]					
	>25	126	31	1.01	[0.64 - 1.61]					
**Tumor size**	T1	27	4	1			**0.007**	1	-	-
	T2	207	42	1.47	[0.53 - 4.09]	0.465		0.99	[0.35 - 2.84]	0.989
	T3	85	30	2.91	[1.03 - 8.27]	0.045		1.92	[0.67 - 5.56]	0.227*
**Clinical nodal status**	N0	141	30	1			**0.317**			
	N1-N2-N3	178	46	1.27	[0.8 - 2]					
**Mitotic Index**	≤22	124	27	1			0.353			
	>22	177	48	1.25	[0.78 - 2]					
**Histology**	NST	291	69	1			0.967			
	others	25	6	0.98	[0.43 - 2.26]					
**Grade**	I-II	40	8	1			0.605			
	III	272	66	1.21	[0.58 - 2.53]					
**Ki67 (%)**	<20	22	4	1			0.492			
	≥ 20	120	32	1.44	[0.51 - 4.07]					
**NAC regimen**	AC	54	12	1			0.418			
	AC-Taxanes	222	57	1.22	[0.65 - 2.27]					
	Others	43	7	0.74	[0.29 - 1.88]					
**TILs**	(continuous)			0,98	[0.98-0.99]		**0,009**			
Post-NAC parameters										
**Nodal Involvment**	0	238	35	1			**<0.001**			
	1-3	49	23	3.9	[2.3 - 6.6]	**<0.001**				
	≥4	32	18	5.27	[2.98 - 9.32]	**<0.001**				
**RCB class**	pCR	123	9	1			**<0.001**	1	-	-
	RCB-I	23	2	1.24	[0.27 - 5.74]	0.784		1.18	[0.25 - 5.51]	0,834
	RCB-II	131	39	4.71	[2.28 - 9.73]	**<0.001**		4	[1.9 - 8.41]	**<0.001**
	RCB-III	42	26	12.09	[5.65 - 25.87]	**<0.001**		8.38	[3.66 - 19.15]	**<0.001**
**LVI**	no	232	41	1			**<0.001**	1	-	-
	yes	55	28	3.69	[2.28 - 5.99]	**<0.001**		1.8	[1.08 - 2.99]	**0.024**
**TILs**	(continuous)			1,01	[0.99-1.01]		0,817			

* p-value T3 *versus* T2 : 0.006

Supplementary Table 6: Association between clinical and pathological pre and post-NAC parameters with overall-free survival (HER2-positive population, univariate and multivariate analysis).

p represents the p-value for the wald test , and p* represents the individual p-value versus reference class. Abbreviations: pCR=pathological complete response; BMI=body mass index ; NST= no special type ; ER=oestrogen receptor; PR=progesterone receptor; NAC=neoadjuvant chemotherapy; AC=anthracyclines; TILs=tumor infiltrating lymphocytes; RCB=residual cancer burden; LVI=lymphovascular invasion

HER2 -positive										
				**Univariate**				**Multivariate**		
**Variable**	**Class**	**Number**	**Events**	**HR**	**CI**	**p***	***p***	**HR**	**CI**	**p**
Pre-NAC parameters										
**Age (years)**	<45	78	5	1			0.5			
	45-55	54	5	1.19	[0.34 - 4.15]					
	>55	44	1	0.33	[0.04 - 2.81]					
**Menopausal status**	pre	114	8	1			0.513			
	post	61	3	0.64	[0.17 - 2.42]					
**BMI class**	19≤BMI≤25	117	4	1			**0.019**	1	-	-
	<19	7	2	11.3	[1.97 - 64.75]	**0.006**		12.86	[2.24 - 73.84]	**0.004**
	>25	52	5	3.43	[0.92 - 12.86]	0.067		2.49	[0.66 - 9.74]	0.18
**Tumor Size**	T1	10	1	1			0.269			
	T2	114	5	0.28	[0.03 - 2.5]					
	T3	52	5	0.7	[0.08 - 6.15]					
**Clinical Nodal Status**	N0	62	3	1			0.622			
	N1-N2-N3	114	8	1.4	[0.37 - 5.28]					
**Mitotic Index**	≤22	112	7	1			0.458			
	>22	59	4	1.61	[0.46 - 5.68]					
**Histology**	NST	170	11	1			0.998			
	others	5	0	0	[0 - Inf]					
**Grade**	I-II	52	5	1			0.373			
	III	121	6	0.58	[0.18 - 1.92]					
**ER status**	negative	77	4	1			0.601			
	positive	99	7	1.39	[0.41 - 4.75]					
**PR status**	negative	111	5	1			0.712			
	positive	60	4	1.28	[0.34 - 4.78]					
**NAC regimen**	AC	4	0	1			0.471			
	AC-Taxanes	152	9	NA	[0 - Inf]					
	Others	20	2	NA	[0 - Inf]					
**TILs**	(continuous)			0,99	[0.95-1.03]		0,732			
Post-NAC parameters										
**Nodal involvment**	0	129	5	1			**0.05**			
	1-3	39	4	2.16	[0.58 - 8.08]	0.253				
	≥4	8	2	7.72	[1.49 - 40.04]	**0.015**				
**RCB class**	pCR	68	1	1			0.146			
	RCB-I	24	0	0	[0 - Inf]	0.998				
	RCB-II	69	7	6.07	[0.75 - 49.48]	0.092				
	RCB-III	15	3	14.25	[1.48 - 137.27]	**0.022**				
**LVI**	no	138	2	1			**<0.001**	1	-	-
	yes	26	7	20.12	[4.17 - 97.13]	**<0.001**		9.74	[2.77 - 34.32]	**<0.001**
**TILs**	**(continuous)**			1,02	[0.98-1.01]		0,328			

Supplementary Table 7: Summary of previous studies comparing prognosis according to RCB score after neoadjuvant chemotherapy (NAC).

Abreviations: CT stands for clinical trial and RA stands for retrospective analysis. Abbreviations: pCR=pathological complete response;

Study	Study type	Number of patients (% of the cohort)				Median Follow-up	5 years RFS whole population	HR whole population	5 y. RFS luminal	5 y. RFS TNBC	5 y. RFS HER2	HR RFS luminal	HR RFS TNBC	HR RFS HER2
		n=	TNBC	HER2	HR+/*HER2* -									
Symmans (2007)<br>*Journal of clinical oncology*	CT<br>T-FAC<br>FAC	382<br>241<br>141	NA	NA	NA	67.3 months<br>104.7 months	RCB-0: 78.2%<br>RCB-I: 83.3%<br>RCB-II: 68.4%<br>RCB-III: 43.3%	HR = 2.50<br>CI [1.70-3.69]<br>p<0.001	NA	NA	NA	NA	NA	NA
Romero (2012)<br>*Annals of Oncology*	CT	151	NA	NA	NA	53.9 months	RCB-0: 81%<br>RCB-I: 66%<br>RCB-II: 66%<br>RCB-III: 35%	RCB- III vs RCB [0-II]= 3.9<br>CI [2336-6401]<br>p = 0.0001	NA	NA	NA	NA	NA	NA
Corben (2013)<br>*Arch. Pathol. Lab. Med.*	CT	56	NA	NA	NA	93 months	RCB-0: 100%<br>RCB-I: 100%<br>RCB-II: 53%<br>RCB-III: 47%	HR = 2.08<br>CI [0,89 - 5,43]<br>p = 0.11	NA	NA	NA	NA	NA	NA
Sheri (2015)<br>*Annals of Oncology*	RA	220	NA	NA	NA	61 months	RCB-0: 91.7%<br>RCB-I: 77.4%<br>RCB-II: 72.7%<br>RCB-III: 38.9%	RCB-0: HR = 1<br>RCB-I: HR = 2.8 ; CI [0.7-11.2] ; p = 0.145<br>RCB-II: HR = 3.0 ; CI [1.1- 8.7] ; p = 0.04<br>RCB-III: HR = 10.36 ; CI [3.7-29.3] ; p < 0.001	NA	NA	NA	NA	NA	NA
Lee (2015)<br>*Plos*	RA	594	**158**<br>(26.6%)	**163**<br>(27.4%)<br>74 HR+/<br>89 HR-	**273**<br>(46%)	37.2 months	NA	NA	NA	NA	NA	p=0.005	p<0.001	p=0.054 for HR+<br>p=0.221 for HR-
Symmans (2017)<br>*Journal of clinical Oncology*	RA<br>T/FAC-1-3<br>FAC<br>H+T/FEC	823<br>132<br>203	**219**<br>(27%)<br>NA<br>0	**103**<br>(12%)<br>NA<br>203 (100%)	**501**<br>(61%)<br>NA<br>0	T/FAC-1-3: 6.8 y to 13.5 y<br>FAC: 16.4 y<br>H+T/FEC: 7.1 y	T/FAC ; FAC ; H+T/FEC<br>RCB-0: 92% ; 100% ; 95%<br>RCB-I: 94% ; 100% ; 81%<br>RCB-II: 80% ; 73% ; 74%<br>RCB-III: 58% ; 66% ; 21%	NA	RCB-0: 88%<br>RCB-I: 100%<br>RCB-II: 87%<br>RCB-III: 70%	RCB-0: 94%<br>RCB-I: 89%<br>RCB-II: 62%<br>RCB-III: 26%	Without / with Trastuzumab:<br>RCB-0: 94% / 95%<br>RCB-I: 89% / 81%<br>RCB-II: 62% / 74%<br>RCB-III: 47% / 21%	HR = 2.28<br>CI [1.76-2.96]	HR = 1.92 ;<br>CI [1.49-2.46]	With trastuzumab<br>HR = 1.8 ;<br>CI [1.26-2.59]
Campbell (2018)<br>*Breast Cancer Research Treatment*	CT	162	**46**<br>(28.4%)	**29**<br>(17.9%)	**87**<br>(53.7%)	6.7 y	RCB-0: 86.5%<br>RCB-I: 84.6%<br>RCB-II: 74.67%<br>RCB-III: 40.5%	RCB-0: HR = 1<br>RCB-I: HR = 4.49 ; CI [1.07-18.79] ; p = 0.04<br>RCB-II: HR = 4.31 ; CI [1.29- 14.36] ; p = 0.02<br>RCB-III: HR = 11.07 ; CI [3.32-36.91] ; p< 0.0001<br>RCB III vs 0/I/II : HR = 3.37 ; IC [1.96-5.8] ; p<0.0001	RCB-0: 87.5%<br>RCB-I: 100%<br>RCB-II: 81.6%<br>RCB-III: 60.9%	RCB-0: 88.2%<br>RCB-I: 100%<br>RCB-II: 58.8%<br>RCB-III: 10%	RCB-0: 83.3%<br>RCB-I: 50%<br>RCB-II: 66.6%<br>RCB-III: 0%	NA	RCB-0: 1<br>RCB-I: not calculated<br>RCB-II: HR=3.65, CI[0.74-18.10]<br>RCB-III: HR=16.92, CI [3.59-79.66]	RCB-0: 1<br>RCB-I: HR= 11.75, CI [(1.21-114.30]<br>RCB-II: HR=6.54, CI [0.73-58.55]<br>RCB-III: HR=22.01, CI [(2.09-232.25]
Pinard (2019)<br>*Breast Cancer Research Treatment*<br>(3 years RFS)	RA	109	**109**<br>(100%)	NA	NA	66.5 months	RCB-I: 91.7%<br>RCB-II: 75.4%<br>RCB-III: 37%	RCB-I: HR = 1<br>RCB-II: HR = 3.66 ; CI [0.84- 16.04] ; p = 0.09<br>RCB-III: HR = 14.3 ; CI [3.31-61.72] ; p = 0.0004	NA	RCB-I: 91.7%<br>RCB-II: 75.4%<br>RCB-III: 37%	NA	NA	RCB-I: HR = 1<br>RCB-II: HR = 3.66 ; CI [0.84- 16.04]<br>RCB-III: HR = 14.3 ; CI [3.31-61.72]	NA
Our study (2019)	RA	717	**319**<br>(44.5%)	**176**<br>(24.5%)	**222**<br>(31%)	99.9 months	RCB-0: 89.9%<br>RCB-I: 89%<br>RCB-II: 67.8%<br>RCB-III: 52.7%	RCB-0: HR = 1<br>RCB-I: HR= 1.38 ; CI [0.56-3.42 ;, p= 0.48<br>RCB-II: HR = 3.05 ; CI [1.88-4.95] ; p < 0.001<br>RCB-III: HR = 5.78 ; CI [3.37-9.93] ; p < 0.001	RCB-0: 72.7 %<br>RCB-I: 88.9 %<br>RCB-II: 69.7 %<br>RCB-III: 55.9 %	RCB-0: 85.36 %<br>RCB-I: 82.6 %<br>RCB-II: 61.8 %<br>RCB-III: 28.6 %	RCB-0: 97 %<br>RCB-I: 95.8 %<br>RCB-II: 72.5 %<br>RCB-III: 60 %	RCB-0: 1<br>RCB-I: HR = 0.37 ; CI [0.06-2.23]<br>RCB-II: HR = 1.13 ; CI [0.35-3.69]<br>RCB-III: HR = 1.58 ; CI [0.49-5.14]<br>p = 0.142	RCB-0: 1<br>RCB-I: HR= 1.23, CI [0.42-3.63]<br>RCB-II: HR=3.1, CI [1.81-5.31]<br>RCB-III: HR=9.01, CI [4.99-16.27]<br>*p<0.0001*	RCB-0: 1<br>RCB-I: HR=1.52, IC [0.14-16.81]<br>RCB-II: HR=10.27, IC [2.39-44.11]<br>RCB-III: HR=16.53, IC [3.33-82.01]<br>*p<0.0001*
