# Supplementary material for: Prognostic value of the Residual Cancer Burden index according to breast cancer subtype: Validation on a cohort of BC patients treated by neoadjuvant chemotherapy
Source: PLoS One. 2020 Jun 24;15(6):e0234191. doi: 10.1371/journal.pone.0234191 (PMC7313974; doi:10.1371/journal.pone.0234191)
Supplement: Attachment — Submitted filename: Response_to_reviewers_PLOS_ONE.docx [file pone.0234191.s002.docx]

**Reviewer #1:**

**This is a well-written manuscript with a worthy study goal.**

We thank reviewer #1 for his interest in our analysis.

**My only comment would be that the TILS data seemed a bit out of place in this study. I would focus this solely on the validation of the RCB index. Data related to LVI and TILS association with RCB is probably saved for a separate manuscript.**

We agree with reviewer #1 that the core of the project was indeed the validation of the RCB score. Our point in adding LVI and TILs to this manuscript was to describe extensively association between RCB and tumor biology. Furthermore, we strongly believe that TILs have become an important feature of BC in the last decade, and we thought that the reader would look for such data.

Finally, we identified an interaction between TILs and RCB, meaning that:

- (i) the prognostic impact of the RCB score varies with the post-NAC TILs levels
- (ii) the prognostic impact of the post-NAC TILS varies with the the RCB score;

Hence, as the value of each variable should be interpreted when taking into account the other one, we consider as clinically relevant to present TILs data.

**Reviewer #2:**

**This article is an independent validation study of the RCB index within the main subtypes of breast cancer and with long-term follow-up. Furthermore, it addresses the independent contributions of lymphovascular invasion (LVI) and tumor infiltrating lymphocytes (TILs) in the residual tumor bed. The methods and results are clear.**

We thank reviewer #2 for his positive comments.

**It appears form the K-M plots that there were few events in the 29 patients with luminal subtype who had pCR or RCB-I (5 RFS events and 2 OS events). It might be that there was insufficient statistical power to observe a difference in that subset - something to discuss with the statistician.**

Reviewer #2 is totally right. Though the association between RCB and survival was highly significant in the whole population, in the TNBC and *HER2*-positive subgroups, the *p*-value failed to reach statistical significance in the luminal subgroup (*p*=0.11). However, both the number of events and the number of patients in the pCR and RCB-I group were low, which clearly resulted in a lack of statistical power to detect a significant association between RCB and survival.

To adress reviewer #2’s question, we performed new analyses while pooling together classes pCR and RCB-I. In the luminal subgroup, the *p*-value for the association between RCB and survival was nearly significant (*p*=0.069).

***Review Figure 1:*** *Association of RCB classes, after having combined pCR and RCB-I classes in a unique class, (pCR/RCB-I to RCB-III) with relapse-free survival (RFS) in: A) whole population (N=717), B) luminal tumors (N=222), C) TNBC (N=319), D) HER2-positive BC (N=176)*

We discussed these statements in the discussion, and we added a paragraph mentioning the pooled meta-analysis of more than 5000 individual RCB data with long-term follow up presented in San Antonio 2019. In this study, RCB was significantly associated with BC outcomes, including in the luminal BC subgroup.

The manuscript was corrected accordingly.

Page 22, lines 11-17:

***“However, a pooled meta-analysis of more than 5000 individual RCB data with long-term follow up was recently presented by Symmans and colleagues***^1^***. In this study, RCB was significantly associated with BC outcomes, even in the luminal BC subgroup. These results are consistent with a lack of power to detect such differences in our data, where the subgroup of patients with luminal subtype who achieved pCR or RCB-1 only included 29 patients, therefore leading to a low number of events.”***

**The LVI result is important and adds new information to the field. I note that a small subset had missing data and so the LVI status was imputed from other variables. It would be helpful to know whether the significance of LVI still holds when the analysis is restricted to the majority subset where LVI status was reported by the pathology review.**

The reviewer is right, and we should document the robustness of the variable “LVI” due to the presence of missing data. As asked by reviewer #2, we conducted new analyses in the very subset of patients whose LVI status was reported by the pathology review (n=648 out of 717). As shown in the tables below, the prognostic significance of LVI still held after multivariate analysis in the whole population, and in TNBC and *HER2*-positive subgroups, but not in the luminal BC subgroup.

***Review Table 1:*** *Multivariate analysis of the association between pre and post-NAC parameters and relapse-free survival in the study population with LVI data available (missing status excluded).*

***Review Table 2:*** *Multivariate analysis of the association between pre and post-NAC parameters and relapse-free survival in the luminal breast cancer population with LVI data available (missing status excluded).*

***Review Table 3:*** *Multivariate analysis of the association between pre and post-NAC parameters and relapse-free survival in the triple negative breast cancer population with LVI data available (missing status excluded).*

***Review Table 4:*** *Multivariate analysis of the association between pre and post-NAC parameters and relapse-free survival in the HER2-positive population with LVI data available (missing status excluded).*

**Some minimal edits to consider:**

1. **Page 6, Pathologic Review, 1st sentence. This sentence could be more clear. Presumably the slides from both the pre-treatment core biopsy and the post-treatment resection specimen were reviewed in all 717 cases.**

We apologize for being unclear. The wording of this sentence was modified for purposes of clarity.

Page 6, Pathologic review, Ist sentence:

***“717 post-NAC pathological pretreatment core needle biopsies and the corresponding post-NAC surgical specimens were reviewed”***

was replaced by:

***“717 pathological pretreatment core needle biopsies and the corresponding post-NAC surgical specimens were reviewed”***

2. **Page 7, last sentence: Was this the Chi-square test?**

It was indeed the Chi-square test.

Page 7, last sentence:

***“Khi2 tests were performed […].”***

was replaced by:

***“Chi-square tests were performed […].”***

**Reviewer #3:**

**It would be helpful to compare your findings to the findings from the Meta-analysis of > 5000 patients incl 5 and 10y EFS presented at SABCS 2019 GS5-01.**

We thank reviewer #3 for his useful advice. At the time we submitted our manuscript (October 2019), we were unable to include the above-mentioned meta-analysis because it had not yet been presented at San Antonio’s Breast Cancer Symposium.

In this meta-analysis, individual RCB data with long-term follow-up (10 years) from more than 5000 patients were collected and pooled. This meta-analysis confirmed the strong prognostic value of the RCB index. In addition, the prognosis impact of the RCB index was found significant in the luminal BCs, consistent with the fact that our study lacked statistical power to detect such difference. We modified our article’s discussion in order to address this issue:

Page 22, lines 11-14:

***“However, a pooled meta-analysis of more than 5000 individual RCB data with long-term follow up was presented by Symmans and colleagues***^1^***. In this study, RCB was significantly associated with BC outcomes, even in the luminal BC subgroup. These results are consistent with a lack of power to detect such differences in our data, where the subgroup of patients with luminal subtype who achieved pCR or RCB-1 only included 29 patients, therefore leading to a low number of events”.***

**It would be helpful to include details about sampling of the breast specimens included. The SOP for RCB was published in 2007. The cohort starts in 2002. How did the sampling of the retrospectively reviewed surgical specimens differ form the RCB SOP? How did this affect calculation of the RCB scores?**

Reviewer #3 raises an important question, which is the evolution of sampling methods during the time period of the cohort. Indeed, as diagnosis tools, surgical biopsies were progressively replaced

by core needle biopsies over time. However, the RCB index only takes into account post-NAC features, *i.e.* features read on post-NAC surgical specimens (invasive tumor cellularity, tumor size, percentage of in situ disease, number of positive lymph nodes, and size of the largest metastasis). In our institution, as well as worldwide to our knowledge, the sampling method of breast cancer surgery was not modified between 2002 and 2012. Hence, we did not observe any clear trend in RCB scores between specimens removed before 2007 and after 2007.

***Review Figure 2:*** *RCB scores repartition between specimens between 2002 and 2012.*

*References :*

**1**. Yau C, van der Noordaa M, Wei J, et al: Residual cancer burden after neoadjuvant therapy and long-term survival outcomes in breast cancer: A multi-center pooled analysis. SABCS Abstract, GS5-01. [Internet]. SABCS Abstr 2019 , 2019[cited 2020 Mar 29] Available from: https://www.abstractsonline.com/pp8/#!/7946/presentation/1923
